# A Case of Wegener's Granulomatosis Presenting with Unilateral Facial Nerve Palsy

**DOI:** 10.1155/2016/9153625

**Published:** 2016-03-24

**Authors:** Roy Ujjawal, Pan Koushik, Panwar Ajay, Chakrabarti Subrata

**Affiliations:** ^1^Department of Neurology, Bangur Institute of Neurosciences, IPGMER, Kolkata 700025, India; ^2^Department of Neurology, King George's Medical University, Lucknow 226003, India; ^3^Department of Endocrinology, IPGMER, Kolkata 700020, India

## Abstract

Wegener's granulomatosis or granulomatosis with polyangiitis is a necrotizing vasculitis affecting both arterioles and venules. The disease is characterized by the classical triad involving acute inflammation of the upper and lower respiratory tracts with renal involvement. However, the disease pathology can affect any organ system. This case presents Wegener's granulomatosis presenting with facial nerve palsy as the first manifestation of the disease, which is rarely reported in medical literature.

## 1. Introduction

Wegener's granulomatosis (WG) is an idiopathic systemic form of vasculitis characterized by the presence of necrotizing granulomas and vasculitis in the upper airways, lower airways, and kidneys. Being a systemic disease, it may additionally involve any organ system. Etiology of this disease is proposed to be autoimmune [1, 2]. Incidence peaks between the ages of 20 and 40 years [2].

## 2. Case Report

**Figure 1:**
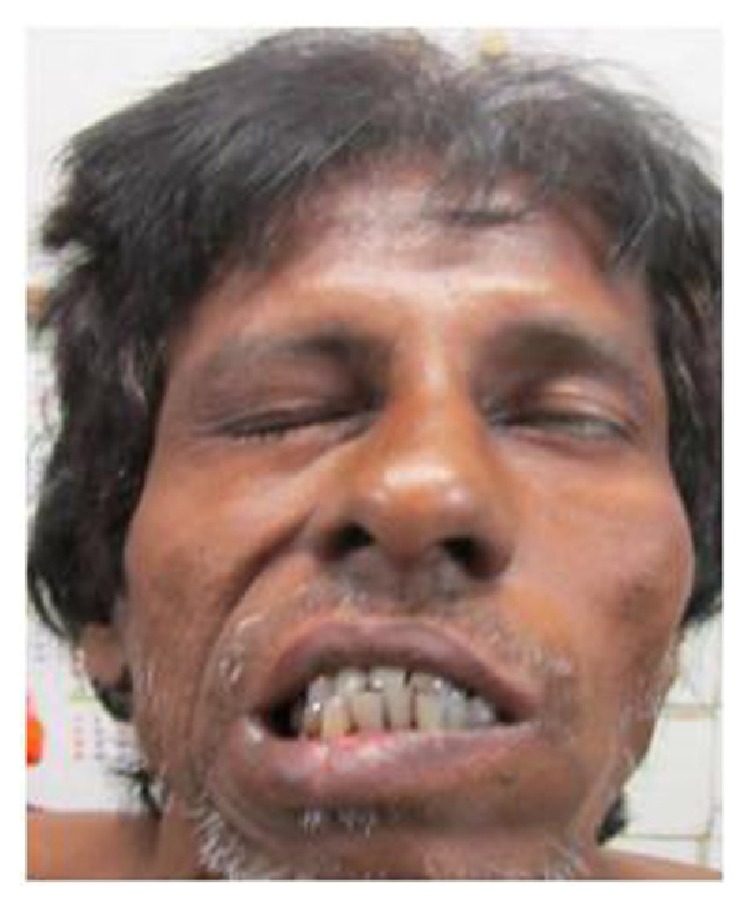
Patient had a left sided lower motor neuron type facial palsy.

A 50-year-old nondiabetic, nonhypertensive male patient was referred from a primary health centre to our institute with a provisional diagnosis of bell's palsy of left side. He had a history of acute onset deviation of angle of mouth to the right and difficulty in closure of left eye 2 days back. There was no history of any preceding fever, local trauma, ear related problem, cough, hemoptysis, chest pain, skin rash, or any hypoesthetic patches over the body. Patient's past history was not significant except for 2-3 episodes of upper respiratory tract infection with persistent rhinorrhea for 2 months in the last one year. General physical examination was notable for bilateral pitting pedal edema. Neurological examination revealed left sided lower motor neuron type of 7th nerve palsy (House-Brackmann grade 5) (Figure 1). The rest of the neurological and systemic examination was normal. Otoscopic examination did not show any abnormality and no hearing loss was detected on audiogram.

**Figure 2:**
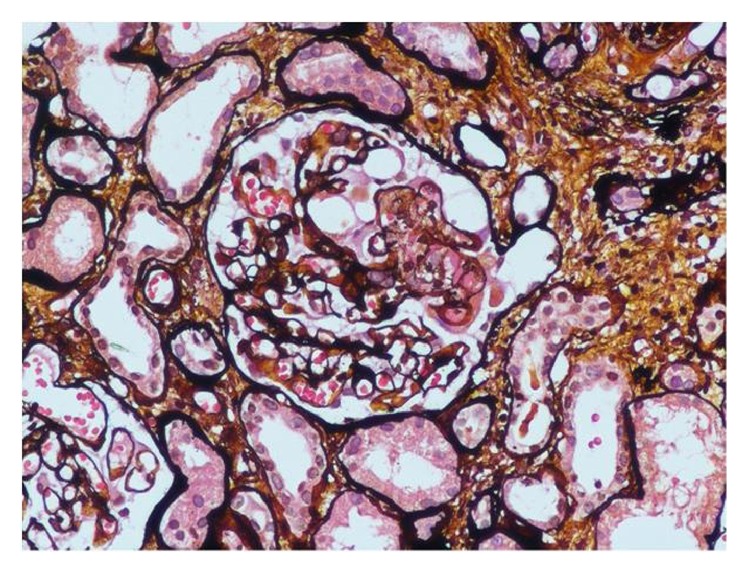
Section of glomerulus with methenamine silver staining showing circumferential cellular crescent with fibrinoid necrosis and leukocytic exudation.

**Figure 3:**
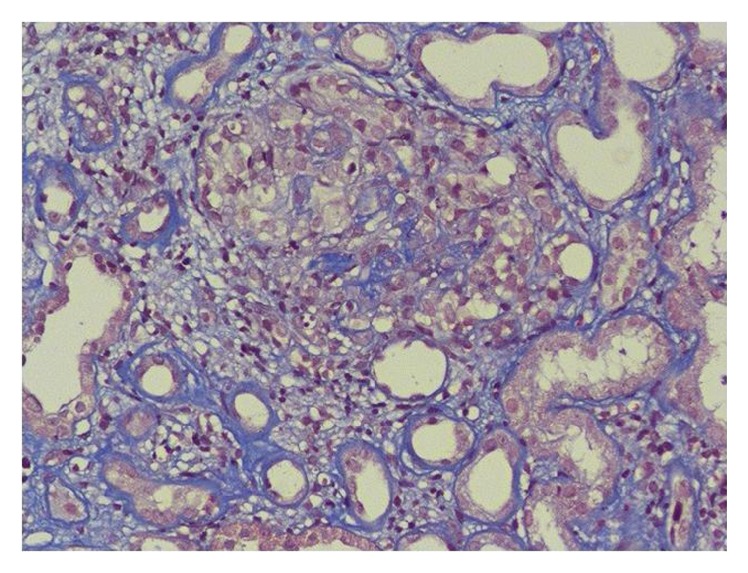
Another section with trichrome staining showing single glomerulus with segmental endocapillary proliferation, fibrinoid necrosis, and breakage of bowman's capsule along with leukocytic exudation.

Laboratory investigations showed mildly elevated erythrocyte sedimentation rate (ESR) (52 mm in 1st hour) and deranged renal function tests (serum urea, 56 mg/dL and creatinine, 2.1 mg/dL). The rest of the routine hematological and biochemical investigations including blood sugar and thyroid profile were normal. Blink reflex study was performed which showed absent ipsilateral R1 and R2 responses on left side stimulation while normally presenting contralateral R2 response. On right side stimulation, ipsilateral R1 and R2 responses were normal while contralateral R2 response was absent. These findings were corroborative of left sided facial nerve palsy. MRI brain did not reveal any abnormality, thus ruling out any structural cause of facial nerve palsy. Viral markers for HIV, Hepatitis B, and Hepatitis C were nonreactive. Chest X-ray was normal. Routine microscopy of urine revealed 3+ proteinuria and red blood cells (RBCs) casts. Accordingly 24-hour urinary protein was sent which came out to be 2.2 g/day. Based on these investigations, patient's history was retrospectively explored. Patient further reported the backdrop of intermittent swelling of feet with mild arthralgia involving both knee and small joints of hands in previous 6 months. On further testing, rheumatoid factor (RF) and antinuclear factor (ANF) were found to be negative. However, c-ANCA was detected positive against proteinase-3 (titre 1 : 640). Kidney biopsy was done (Figures 2 and 3) which showed features suggestive of pauci-immune crescentic glomerulonephritis (PICGN). Based on these investigations, patient was diagnosed as a case of Wegener's granulomatosis.

Treatment was started (1 week after presentation) according to EUVAS (European Vasculitis Study Group) protocol for the management of primary small and medium vessel vasculitis. Initially he received 3 pulses of daily intravenous methyl-prednisolone (1 gm) followed by intravenous cyclophosphamide at a dose of 15 mg/kg every 2 weeks for the first 3 pulses, followed by infusions every 3 weeks for the next 3 pulses. We also put him on a remission-maintenance therapy with a combination of low-dose glucocorticoid (0.25 mg/kg) and azathioprine (2 mg/kg).

Patient's clinical features were closely followed and a urine analysis performed 15 weeks after starting immunosuppressive therapy revealed only 1+ proteinuria (260 mg/24 hours) without any RBCs. By this time patient improved significantly from his systemic involvement with normalization of renal function parameters along with complete recovery from facial nerve paralysis.

## 3. Discussion

Wegener's granulomatosis or granulomatosis with polyangiitis is an uncommon, systemic disease that mainly affects the head and neck region, the lungs, and the kidneys. Besides, a significant proportion of patients present with peripheral nervous system (PNS) involvement in the form of mononeuritis multiplex due to vasculitis or with central nervous system (CNS) involvement due to infiltrating granulomatous lesions (10–45%) [3]. The common presenting manifestations of the disease are recurrent sinusitis, nasal ulceration with discharge, and symptoms of lower respiratory tract infection. Some of the patients may present with hematuria and peripheral edema due to kidney involvement. Our patient atypically presented with left sided lower motor neuron (LMN) facial palsy. In absence of any systemic symptoms or signs, initially we thought about common possibilities of LMN facial nerve palsy including bell's palsy, herpes infection, Guillain-Barre syndrome, and pontine small vessel stroke. MRI brain, turning out to be normal, ruled out the structural brain stem lesion. However, patient was evaluated further in view of peripheral edema and was established to have evidence of kidney disease on urine examination, further confirmed by renal biopsy. Positive serum c-ANCA suggested the diagnosis of Wegener's granulomatosis, featuring with pauci-immune crescentic glomerulonephritis. Neurological involvement is commonly seen in Wegener's granulomatosis. Reviewing the current literature, a study from the Mayo Clinic found that almost 30% of 324 patients had neurological involvement [4]. The cranial nerves most commonly affected were II, VI, and VII while IX, X, and XII were less commonly affected. Notwithstanding the common neurological involvement, cranial neuropathy as the first manifestation of the disease preceding other organs involvement is uncommon.

Unilateral facial nerve palsy in our case was a probable manifestation of vasculitis leading to ischemic cranial neuropathy. Alternatively, the cranial neuritis could be a result of immune-mediated inflammatory process. Cadoni et al. [5] substantiated the pathological role of antiendothelial cell antibodies (AECAs) in WG and hypothesized them to be useful for the early diagnosis of WG. However, we could not perform AECAs analysis due to unavailability of this facility at our institute. Its diagnostic role in WG appears to be promising though and, in our opinion, further research work is needed to support the same.

Isolated facial cranial neuritis as the first presentation of the disease is rarely reported. Although, there have been a few reports of bilateral facial nerve palsy. Nikolaou et al. reported a case of a young female who presented with bilateral serous otitis media followed by bilateral sensorineural hearing loss and bilateral facial nerve palsy [6]. Wegener's disease presenting as bilateral facial nerve palsy has also been reported by Ferri et al. [7].

Prognosis of neuropathies associated with Wegener's granulomatosis depends mainly on early diagnosis and prompt initiation of treatment [8–10].

## 4. Conclusion

Wegener's granulomatosis is an autoimmune disease and can be lethal if left untreated. Long term remission can be achieved in up to 90% of the cases with the help of therapeutic agents like steroids and other immunosuppressant drugs such as cyclophosphamide, azathioprine, and methotrexate. The presentation of Wegener's granulomatosis can be varied and the patients may initially present as an isolated cranial neuritis. Physicians should be aware of the atypical and varied presentations of this disorder, which may thus help in early diagnosis and initiation of treatment. Lower motor neuron type of facial paralysis is not always synonymous with bell's palsy and secondary causes should always be ruled out by a thorough clinical examination and a comprehensive investigation panel.

## Additional Points

Wegener's granulomatosis can present with isolated cranial neuritis as the first manifestation of the disease. Physicians need to be aware of Bell's palsy as the presenting feature of this disease, so as to ensure the timely diagnosis and early treatment.
